# Effectiveness of two community-based strategies on disease knowledge and health behaviour regarding malaria, diarrhoea and pneumonia in Ghana

**DOI:** 10.1186/s12889-017-4964-6

**Published:** 2017-12-12

**Authors:** Blanca Escribano-Ferrer, Margaret Gyapong, Jane Bruce, Solomon A. Narh Bana, Clement T. Narh, Naa-Korkor Allotey, Roland Glover, Charity Azantilow, Constance Bart-Plange, Isabella Sagoe-Moses, Jayne Webster

**Affiliations:** 1 Disease Control Department, London School of Hygiene and Tropical Medicine, London, UK; 2 Institute of Health Research, University of Health and Allied Sciences, Ho, Volta Region Ghana; 3 Dodowa Health Research Center, Ghana Health Service, Dodowa, Ghana; 4 School of Public Health, University of Health and Allied Sciences, Ho, Volta Region Ghana; 5 National Malaria Control Programme, Ghana Health Service, Accra, Ghana; 6 Reproductive and Child Health Department, Ghana Health Service, Accra, Ghana

**Keywords:** Home- based care, Integrated community case management (iCCM), Integrated Management of Childhood Illness (IMCI), Malaria, Diarrhoea, Pneumonia, Children under- five, Prevention, Health promotion

## Abstract

**Background:**

Ghana has developed two community-based strategies that aim to increase access to quality treatment for malaria, diarrhoea and suspected pneumonia, and to improve household and family practices: integrated Community Case Management (iCCM) and Community-based Health Planning and Services (CHPS). The objective of the study was to assess the effectiveness of iCCM and CHPS on disease knowledge and health behaviour regarding malaria, diarrhoea and pneumonia.

**Methods:**

A household survey was conducted two and eight years after implementation of iCCM in the Volta and Northern Regions of Ghana respectively, and more than ten years of CHPS implementation in both regions. The study population included 1356 carers of children under- five years of age who had fever, diarrhoea and/or cough in the two weeks prior to the interview. Disease knowledge was assessed based on the knowledge of causes and identification of signs of severe disease and its association with the sources of health education messages received. Health behaviour was assessed based on reported prompt care seeking behaviour, adherence to treatment regime, utilization of mosquito nets and having improved sanitation facilities, and its association with the sources of health education messages received.

**Results:**

Health education messages from community-based agents (CBAs) in the Northern Region were associated with the identification of at least two signs of severe malaria (adjusted Odds Ratio (OR) 1.8, 95%CI 1.0, 3.3, *p* = 0.04), two practices that can cause diarrhoea (adjusted OR 4.7, 95%CI 1.4, 15.5, *p* = 0.02) 0and two signs of severe pneumonia (adjusted OR 7.7, 95%CI2.2, 26.5, *p* = 0.01)-the later also associated with prompt care seeking behaviour (*p* = 0.04). In the Volta Region, receiving messages on diarrhoea from CHPS was associated with the identification of at least two signs of severe diarrhoea (adjusted OR 3.6, 95%CI 1.4, 9.0), *p* = 0.02). iCCM was associated with prompt care seeking behaviour in the Volta Region and CHPS with prompt care seeking behaviour in the Northern Region (*p* < 0.5).

**Conclusions:**

Both iCCM and CHPS were associated with disease knowledge and health behaviour, but this was more pronounced for iCCM and in the Northern Region. HBC should continue to be considered as the strategy through which community-IMCI is implemented.

**Electronic supplementary material:**

The online version of this article (10.1186/s12889-017-4964-6) contains supplementary material, which is available to authorized users.

## Background

In the 1990s the World Health Organisation (WHO) and the United Nations International Children’s Emergency Fund (UNICEF) developed the integrated management of childhood illness (IMCI) to reduce the morbidity and mortality of children under- five [1]. The IMCI strategy has 3 aims: (i) to improve the management of childhood illness through an integrated approach (as opposed to a disease-specific approach), (ii) to strengthen the health system through the training human resources and ensuring the availability of drugs and (iii) to improve family and community health practices through better counselling caretakers in outpatient settings and partnering with the community in home settings to promote appropriate care seeking behaviours, improved nutrition and preventative care [2–11]. Several years later, recognising the challenge of access and prompt access to health care services and the importance of self-medication practices, WHO and the Roll Back Malaria Initiative indicated that in settings with limited access to health facilities, diagnosis and treatment should be provided at the community level through community case management [12–15]. Integrated community case management (iCCM) involves treating sick children with pre-packaged drugs dispensed by individuals selected by the community and trained by health care providers, thereby enhancing the potential to reduce inequities in access to quality drugs. In addition, the iCCM strategy shares the aim of IMCI in improving family and community health practices.

Ghana adopted the IMCI and iCCM strategies and now implements two main community-based strategies to reduce morbidity and mortality of children under-five: the community-based health planning services (CHPS) and the iCCM.

The CHPS strategy started in Ghana in 1994 as a pilot study. It is based upon a basic health facility known as a community health compound. Health services are provided by a resident community health nurse, or community health officer, who also does a 90 day cycle visiting the communities she/he serves at least once within that period [16]. CHPS implement the IMCI strategy (now called IMNCI which is Integrated Management of Neonatal and Childhood Illnesses [17]). Health promotion and disease prevention messages are delivered by nurses to carers in the waiting area of health facilities (group education sessions), during consultation (individual sessions) and during outreach visits (individual or group sessions). Nurses from CHPS also include information, education and communication (IEC) activities in their monthly reports to the district.

The iCCM (then called HBC) started in Ghana in 1999 as a pilot study to treat malaria cases at the community level. In 2009 pneumonia and diarrhoea were included [16, 18]. iCCM was defined as prevention, early case detection and prompt and appropriate treatment of fevers, pneumonia and diarrhoea in the community. The iCCM strategy corresponds to the lowest level of health care delivery in Ghana and it is implemented through community-based agents (CBAs). CBAs conduct IEC activities including the promotion of insecticide treated nets (ITN), intermittent preventive treatment for malaria prevention in pregnant women (IPTp), indoor residual spraying (IRS) and hygiene measures. According to policy, CBAs are given a stock of IEC materials and conduct health education activities in the community on a monthly basis. Health education activities are supervised by health facility staff. CBAs include IEC activities conducted in their monthly reports to health facilities. IEC activities may target individuals or groups, such as those in churches, mosques or prayer camps. In 2014, 163,468 children under-five were checked for illness and 503,550 IEC activities were conducted nationally (source: DHIMS2-District Health Information Management System 2).

The type of messages delivered by iCCM and CHPS are very similar, with the CHPS messages being more comprehensive, including child growth, vaccine promotion, appropriate care for children living with HIV and full antenatal care promotion.

After several years of national implementation, it is important to know how effective iCCM and CHPS are at delivering preventive and curative health services, known to contribute to reducing the morbidity and mortality of children under-five. Studies that have looked at the efficacy of the iCCM in Ghana mostly focused in a few districts, looked particularly at the management of malaria HBC, neglecting the preventive component and were conducted in a more “controlled” context [19–23]. We reported on the utilization, effectiveness, cost-effectiveness and users’ satisfaction of the curative component of iCCM and CHPS in two regions of Ghana [16, 24]. In this paper we report on the effectiveness of the preventive component of iCCM and CHPS on family and community practices.

## Methods

### Study sites

Ghana has ten regions, all implementing iCCM and CHPS. The study took place in the Volta and Northern Regions.

The iCCM in the Volta Region started in 2012 mainly with the support of the Global Fund and targeted only rural districts for the iCCM implementation. While only including a curative component for malaria, iCCM in Volta Region provided preventive and promotive messages for malaria, diarrhoea and pneumonia. Volta Region had a population of 1,901,179 habitants, 17 districts, 674 CHPS and 920 CBAs in 2014. Malaria prevalence was 17%, diarrhoea prevalence was 7.6% and suspected pneumonia prevalence was 2.1% in children under- five [25]. The rural population corresponded to 66% of the total population. Two rainfall seasons occur in the middle and coastal belts. One major season is in April/July with a peak in June and one minor season is in September/November with a peak in October. The north of Volta Region has one rainy season - May to October with a peak in August.

The iCCM in the Northern Region started in 2006 mainly with the support of UNICEF, targeted all communities and provided treatment and preventive and promotive messages for malaria, diarrhoea and pneumonia. It had a population of 2,479,461 habitants, 20 districts, 210 CHPS and 5000 CBAs in 2014. Malaria prevalence was 48%, diarrhoea prevalence was 21.4% and suspected pneumonia prevalence was 6.3% in children under five (163). The rural population corresponded to 70% of the total population. In the north, the rainy season begins in May and ends in October (164). Climatically, religiously, linguistically, and culturally, the Northern Region differs greatly from the politically and economically dominating regions of central and southern Ghana, and it is similar to the two other northern regions (Upper East and Upper West).

The CHPS strategy started in 2004 in both regions and is uniform across regions of the country.

### Study design and sampling procedures

Data was collected from a cross sectional study aimed at assessing the utilisation, the effectiveness of the curative and preventive components, the satisfaction and the cost-effectiveness of iCCM and CHPS [16]. This was an observational study, post implementation and without controls. The effectiveness of the delivery of the curative and preventive components was assessed against national guidelines and standard definitions. The study population were carers of children under- five who had a fever, cough and or diarrhoea in the last two weeks.

The Volta and the Northern Region were purposely selected. Criteria for selecting the Volta and the Northern Regions are described in Ferrer et al. [16]. The first criteria was the inclusion of a region implementing iCCM but providing only treatment for malaria and a Region providing treatment for malaria, diarrhoea and pneumonia, to have a better picture of the iCCM strategy in Ghana. The second criteria was different implementation performance levels, based on the routine information system. As mentioned above, the iCCM strategy in the Volta Region provides only treatment for malaria while in the Northern Region treatment for the three diseases are provided. Based on the monthly activities reported through the routine monitoring information system (District Health Information System-DHIMS2), the NMCP had some concerns on the low number of iCCM activities conducted (preventive and curative) while being satisfied with the number of activities reported in the Volta Region. One “good” and one “bad” performing Region were selected to contrast results with those of DHIMS II.

A stratified three stage cluster survey was conducted in the Volta and Northern Regions. In order for the sample to be representative of the whole region, whilst being logistically feasible, regions were divided into 3 areas. From each area, 2 districts and from each district four clusters were selected using probability proportional to size. Then, from each cluster 27 households were planned to be selected, making a total of 648 in each region. To select the districts (first stage) the list of districts implementing iCCM (all districts implement the CHPS strategy) with its population was used. According to the NMCP, from the 24 districts in the Volta Region, only 8 were targeted for the implementation of iCCM and were included in the sampling. In the Northern Region all districts and communities were included in the sampling, as iCCM was implemented everywhere. To select the clusters (second stage) the list of communities implementing iCCM with its population was used. Then, households with children under- five that had fever, diarrhea or cough in the last two weeks prior to the interview were randomly selected in each cluster using a modified expanded programme on immunizations sampling technique (third stage) [26].

Carers of children presenting with fever, diarrhoea or cough in the previous two weeks were asked by the interviewer about their exposure to disease prevention and health promotion messages in the year previous to the survey, the source of the messages, their knowledge about the causes of malaria, diarrhoea and acute respiratory infections, the identification of signs of severe disease, treatment seeking behaviour when the child was sick, adherence to the treatment prescribed and other health behaviours and household characteristics. These were considered to be questions that were valid for assessing both iCCM and CHPS prevention strategies in the Volta and Northern Regions. To attribute the outcomes to the delivery strategies under study, the questionnaire included different question such us “where did you seek care”, “from where did you receive the drug”, “from where did you receive health promotion and preventive messages”, as used previously in a similar study in Ghana [27]. The exposures of interest were CBA and CHPS disease prevention and health promotion messages and type of provider sought (CBA and CHPS) when child was sick. Outcomes explored were disease knowledge and health behaviour described in Additional file 1: Table S1. Other explanatory variables or predictors explored included age of respondents, education of respondents, socioeconomic status, other source of messages and other providers sought (Additional file 1: Table S2).

Data collection was done using a structured questionnaire. The questionnaire was carefully designed and tested to ensure its validity and reliability to address the research questions, although it is likely that the responses on knowledge are more valid than those on practice as this is reported and not observed practice. To ensure that the data collected was of good quality, supervisors conducted checks for errors on the questionnaires, daily. This exercise was followed by a meeting with field workers to give feedback on issues found and discuss how to solve the problems identified.

### Study definitions

For the purposes of this study, effectiveness of the preventive component on family and community practices relates to disease knowledge and health behaviour.

Indicators chosen to measure disease knowledge were carers of under- five children being able to: (i) identify the cause and causal practices of malaria, diarrhoea and pneumonia and (ii) identify signs of severe disease. Indicators chosen to measure health behaviour were: (iii) children under- five sleeping under an ITN/LLIN, (iv) improved sanitation facilities in the household, (v) promptness in seeking care and (vi) adherence to treatment.

These indicators were appropriate to measure the effectiveness of the preventive component of the iCCM and CHPS strategies because (i) they could assess achievement of the objectives of the iCCM and CHPS; (ii) they were comparable with indicators used in other studies [28–30], (iii) they were feasible to obtain using a survey questionnaire and (iv) they allow attribution of outcomes to exposure to the different strategies based on the source of health education messages. In addition, these indicators are proxy indicators of child survival, child growth and development [31–36].

### Data management and analysis

Data were double entered and validated using EpiData 3.1. Thereafter, data processing and analysis was done using STATA 12. Initial data examination and point estimates were obtained using tabulations. All estimates were adjusted for the design of the survey, Pearson’s chi-square was used to test for associations and survey logistic regression was used to obtain adjusted estimates.

To explore the association between disease knowledge and health behavior, and potential predictors (Additional file 1: Table S1 and Table S2), the crude Odds Ratio (OR) was obtained using unadjusted model for a single variable models, and the adjusted OR using the adjusted model for multivariable. Disease knowledge outcomes included were identification by carers of (i) mosquitos as vectors of malaria transmission, (ii) at least 2 signs of severe malaria, (iii) at least 2 practices that can cause diarrhoea, (iv) at least 2 signs of severe diarrhoea, (v) microorganisms as the cause of suspected pneumonia, (vi) not vaccinating children as a practice that can cause pneumonia and (vii) 2 signs of severe pneumonia. Potential predictors explored are described in Additional file 1: Table S2. To explore the potential association between disease knowledge and predictors, firstly, the association of each potential predictor (adjusted only for districts) with the outcome was estimated. All factors whose association reached significance at *p* < 0.1 were included in a multivariable analysis. All factors that remained significantly associated with the outcome (*p* < 0. 05) in this model were retained in the final model.

The same methodology was used to explore potential predictors of children sleeping under mosquito nets. Potential predictors were explored in the unadjusted model (adjusted only for districts). All factors whose association reached significance at *p* < 0.1 were included in a multivariate analysis. All factors that remained significantly associated with the outcome (*p* < 0. 05) in this model were retained in the final model.

To analyse potential predictors of having an improved sanitation facility, potential predictors (Additional file 1: Table S2) were explored in the unadjusted and adjusted analysis using the same methodology.

## Results

A total of 1356 interviews were conducted in the Volta and Northern Regions (685 and 671 respectively). The response rate was very high with no refusals to participate. Respondents were mainly female farmers aged between 20 and 29 years old, married or co-habiting. Respondents in the Northern Region were less educated than those in the Volta Region (Additional file 1: Table S3).

Using the year preceding the survey as a point of reference, a higher proportion of carers reported receiving messages relating to malaria than those relating to diarrhoea or respiratory infections across the two regions. The proportion of carers who reported receiving malaria education messages (including information on any of: how it is transmitted, how to avoid it, what are the symptoms and what to do when sick) was 83.8% (95%CI 57.3, 95.2) and 89.0% (95%CI 80.9, 93.6) in the Volta and Northern Region, respectively (Table 1).

**Table 1:** Sources of malaria, diarrhoea and respiratory information and education messages in the Volta and Northern Regions

Health education	Volta Region		Northern Region	
	N	% (95%CI)^a^	N	% (95% CI)^a^
Receiving messages on malaria	576/671	83.8	575/685	88.8
Sources of malaria messages				
From CBAs	74	18.5 (4.9, 50.0)	43	8.5 (3.2, 20.4)
From CHPs nurses	118	31.2 (11.5, 61.3)	182	26.8 (14.9, 43.3)
From HC nurses	195	21.7 (15.7, 29.0)	131	22.3 (11.5, 38.7)
From hospital nurses	142	25.5 (10.2, 50.7)	75	13.7 (7.2, 24.7)
Outreach clinic	23	4.0 (0.6, 22.1)	0	0
From radio	201	34.3 (23.6, 46.9)	254	44.5 (21.1, 70.6)
From neighbours	47	12.8 (6.4, 23.8)	242	45.8 (40.5, 51.2)
Family member	41	4.2 (1.3, 12.9)	160	33.2 (22.6, 45.9)
Friends	23	3.8 (2.0, 7.1)	109	18.5 (13.0, 25.7)
From TV	66	8.6 (2.0, 29.7)	24	2.0 (0.7, 5.4)
From posters	11	1.0 (0.1, 8.7)	2	0.1 (0.007, 1.7)
Receiving messages on diarrhoea	496/671	73.65	491/685	79.6
Sources of diarrhoea messages				
From CBAs	55	15.0 (4.7, 38.9)	33	7.4 (2.7, 18.8)
From CHPs nurses	107	33.3 (12.5, 63.6)	153	28.8 (16.3, 45.6)
From HC nurses	161	18.1 (9.1, 32.9)	105	20.7 (10.2, 37.3)
From hospital nurses	106	19.0 (5.1, 50.6)	62	11.5 (5.4, 22.7)
Outreach clinic	23	5.1 (0.7, 27.7)	0	0
From neighbours	39	8.2 (2.3, 25.2)	208	46.4 (38.6, 54.3)
Family member	39	4.4 (1.4, 13.2)	150	31.5 (20.8, 44.6)
Friends	27	9.2 (3.0, 24.7)	116	20.8 (15.7, 27.0)
From radio	180	32.3 (23.3, 42.7)	191	37.9 (18.0, 63.0)
From TV	61	9.0 (1.7, 35.0)	19	4.1 (1.1, 13.3)
From posters	9	0.9 (0.1, 7.7)	9	0.8 (0.05, 11.9)
Receiving messages on respiratory infections	345/669	57.1	400/683	66.1
Sources of respiratory infections messages				
From CBAs	34	18.2 (3.3, 59.2)	21	4.6 (1.9, 10.5)
From CHPs nurses	73	31.2 (12.3, 59.6)	99	21.7 (13.8. 32.5)
From HC nurses	99	19.0 (12.6, 27.8)	91	21.4 (9.1, 42.3)
From hospital nurses	78	19.6 (5.0, 53.1)	49	12.1 (3.7, 33.2)
Outreach clinic	13	6.0 (0.7, 34.6)	0	0
From neighbours	34	8.4 (2.0, 29.2)	162	41.1 (33.9, 49.2)
Family member	40	8.0 (1.7, 30.1)	119	28.6 (20.7, 38..1)
Friends	16	6.8 (2.6, 16.5)	98	21.8 (13.4, 33.4)
From radio	127	30.5 (20.1, 43.4)	138	34.1 (19.8, 52.0)
From TV	35	5.3 (1.2, 20.3)	10	0.8 (0.2, 3.5)
From posters	2	0.0 (0.004, 0.4)	8	1.1 (0.0, 17.4)

^a^Weighted estimates

The proportion of carers in the Volta Region receiving malaria education messages from CBAs and CHPS were 18.5% (95%CI 4.9, 50.0) and 31.2% (95%CI 11.5, 61.3) respectively while in the Northern Region this was 8.5% (95%CI 3.2, 20.4) and 26.8% (95%CI 14.9, 43.3) respectively (Table 1).

### Carers’ knowledge of malaria, diarrhoea and respiratory infections

Most of the carers identified mosquitoes as malaria vectors (more than 90% in both regions). However, those that correctly identified severe signs of malaria were much fewer (approximately 22% and 29% in the Volta and Northern Region respectively) and with greater variation among clusters in the Northern Region (Table 2).

**Table 2:** Carer’s disease knowledge in the Volta and Northern Regions

	Volta Region		Northern Region	
Indicator	*N*	% (95% CI)^a^	*N*	% (95% CI)^a^
Identifying mosquito as malaria vector	645	96.8 (90.4, 98.9)	626	91.0 (84.5, 94.9)
Identifying at least 2 signs of severe malaria	135	21.9 (13.0, 34.3)	217	28.5 (13.6, 50.2)
Identifying at least 2 practices that can cause diarrhoea	229	35.8 (29.6, 42.5)	394	52.6 (41.2, 63.8)
Identifying at least 2 signs of severe diarrhoea	185	30.3 (21.4, 41.0)	336	47.4 (38.6, 56.3)
Identifying microorganism as cause of respiratory infection	92	21.1 (12.4, 33.6)	246	36.8 (23.4, 52.5)
Identifying vaccination as practice to prevent respiratory infections	5	0.2 (0.01, 3.8)	73	12.0 (9.1, 15.5)
Identifying 2 signs of severe pneumonia	52	8.7 (4.4, 16.5)	184	25.5 (19.7, 32.4)

^a^Weighted estimates

Only 36% and 53% of carers in the Volta and the Northern Regions identified at least 2 practices that can cause diarrhoea. Causes and practices leading to respiratory infections were less known to carers than those of malaria and diarrhoea.

### Predictors of carer’s knowledge of malaria, diarrhoea and respiratory infections

Different factors were found to be associated to disease knowledge in the Volta and the Northern Region. In the Volta Region, the multivariate analysis showed that receiving malaria education messages (from any source) was associated with the identification of mosquitos as malaria vectors [adjusted OR 15.6 (95%CI 3.2, 75.9), *p* = 0.01] (Table 3). Receiving health education and health promotion messages from CBAs was not a predictor of disease knowledge across any of the outcomes explored (Table 3). However, carers receiving diarrhoea messages from CHPS were more likely to identify at least two signs of severe diarrhoea [adjusted OR 3.6 (95% CI 1.4, 9.0); *p* = 0.02]. On the contrary, in the Northern Region receiving messages from CHPS, was not found to be associated with disease knowledge. However, carers receiving health messages from CBAs was significantly associated with the identification of at least (i) two signs of severe malaria [adjusted OR 1.8 (95%CI 1.0, 3.3); *p* = 0.04], (ii) two practices that can cause diarrhoea [adjusted OR 4.7 (1.4, 15.5); *p* = 0.02] and (iii) two signs of severe pneumonia [adjusted OR 7.7 (95%CI 2.2, 26.5); *p* = 0.01] (Table 3).

**Table 3:** Predictors for disease knowledge in the Volta and Northern Regions

	VOLTA REGION				NORTHERN REGION		
Predictors	n/N (%)	Adjusted OR (95% CI)	*P* value	Predictors	n/N (%)	Adjusted OR (95% CI)	*P* value
IDENTIFYING MOSQUITOS AS MALARIA VECTORS				IDENTIFYING MOSQUITOS AS MALARIA VECTORS			
Receiving malaria messages (any source)				Receiving malaria messages from neighbours			
No	79/95 (86.3)	1	0.01	No	292/333 (85.5)	1	0.05
Yes	566/576 (98.8)	15.6 (3.2, 75.9)		Yes	238/242 (99.0)	16.2 (0.9, 268)	
IDENTIFYING AT LEAST 2 SIGNS OF SEVERE MALARIA				IDENTIFYING AT LEAST 2 SIGNS OF SEVERE MALARIA			
Receiving malaria messages from friends				Receiving malaria messages from CBA			
No	121/553 (21.2)	1	0.01	No	154/532 (25.5)	1.0	0.04
Yes	2/23 (0.5)	0.01 (0.001, 0.1)		Yes	22/43 (50.6)	1.8 (1.0, 3.3)	
				Receiving malaria messages from family			
				No	140/415 (32.9)	1.0	0.04
				Yes	36/160 (17.3)	0.4 (0.2, 0.9)	
				Receiving malaria messages from radio			
				No	52/321 (13.4)	1.0	0.04
				Yes	124/254 (45.5)	4.3 (1.0, 17.6)	
IDENTIFYING AT LEAST 2 CAUSES OF DIARRHOEA TRANSMISSION				IDENTIFYING AT LEAST 2 CAUSES OF DIARRHOEA TRANSMISSION			
				Receiving diarrhoea messages from CBA			
				No	262/458 (52.6)	1.0	0.02
				Yes	26/33 (76.5)	4.7 (1.4, 15.5)	
				Receiving diarrhoea messages from friends			
				No	189/375 (47.3)	1.0	0.008
				Yes	99/116 (81.6)	6.9 (2.6, 18.3)	
				Receiving diarrhoea messages from radio			
				No	145/300 (43.1)	1.0	0.009
				Yes	143/191 (72.9)	4.5 (2.0, 10.2)	
IDENTIFYING AT LEAST 2 SIGNS OF SEVERE DIARRHOEA				IDENTIFYING AT LEAST 2 SIGNS OF SEVERE DIARRHOEA			
Receiving diarrhoea messages from CHPS				Receiving diarrhoea messages from neighbours			0.05
No	97/389 (25.7)	1	0.02	No	120/283 (35.5)	1.0	
Yes	56/107 (44.7)	3.6 (1.4, 9.0)		Yes	144/208 (69.8)	3.3 (0.9, 11.4)	
IDENTIFYING MICROORGANISM (INFECTIONS) AS ARI CAUSE				IDENTIFYING MICROORGANISM (INFECTIONS) AS ARI CAUSE			
Receiving ARI messages from hospital				Receiving ARI messages from friends			
No	64/269 (30.1)	1	0.01	No	75/303 (29.1)	1	0.05
Yes	12/78 (14.8)	0.2 (0.1, 0.6)		Yes	77/116 (58.5)	9.1 (4.4, 18.6)	
Receiving ARI messages from leaflets				Receiving ARI messages from posters			
No	74/344 (27.1)	1	0.002	No	147/393 (38.6)	1	0.05
Yes	2/3 (75.0)	19.4 (7.4, 50.7)		Yes	5/8 (70.8)	8.5 (0.9, 76.9)	
IDENTIFYING AT LEAST 2 SIGNS OF SEVERE PNEUMONIA				IDENTIFYING AT LEAST 2 SIGNS OF SEVERE PNEUMONIA			
Receiving ARI messages from TV				Receiving ARI messages from CBA			
No	35/312 (6.8)	1	0.02	No	127/380 (32.9)	1.0	0.01
Yes	5/35 (48.1)	9.4 (1.9, 45.6)		Yes	15/21 (69.3)	7.7 (2.2, 26.5)	
				Receiving ARI messages from friends			
				No	81/303 (26.8)	1.0	0.006
				Yes	61/98 (62.3)	5.3 (2.4, 11.6)	

While receiving health education messages from hospitals showed conflicting results in the Volta Region [adjusted OR 0.2 (95% CI 0.1, 0.6), *p* = 0.01], no association was found between disease knowledge and messages received from hospitals in the Northern Region.

Friends, neighbours and family members were not only an important source of disease prevention and health promotion messages in the Northern Region but were also associated with disease knowledge. This association was stronger with regards to messages from friends, which were associated with the identification of (i) at least two practices that cause diarrhoea [adjusted OR 6.9 (95%CI 2.6, 18.3); *p* = 0.008]; (ii) microorganism as cause of ARI [adjusted OR 9.1 (95%CI 4.4, 18.6); *p* = 0.05] and (iii) two signs of severe pneumonia [adjusted OR 5.3 (95%CI 2.4, 11.6); *p* = 0.006].

Mass media and small media were also associated with some of the outcomes explored in the Volta and the Northern Region. No predictors of the identification of children vaccination as a practice to reduce acute respiratory infections were found in the Northern Region. The analysis was not conducted in the Volta Region due to the low numbers (only 5 carers believed that not vaccinating their children can cause respiratory infections).

### Carers’ health behaviour

The proportion of carers that reported to have a mosquito net hanging over any (at least 1) bed in the household was 43.0%, (95%CI 35.0, 51.3) in the Volta Region and 70.1% (95%CI 45.2, 87) in the Northern Region. However, 80% of carers of both regions reported that their children under- five slept under a mosquito net the night before the survey (80.4%, 95%CI 69.4, 88.1 and 79.2%, 95%CI 57.5, 91.5 in the Volta and Northern Region, respectively). Almost all carers interviewed in both regions reported that they would accept to have their house sprayed if offered (Table 4).

**Table 4:** Carer’s health behaviour in the Volta and Northern Regions

	Volta Region		Northern Region	
Indicator	*N*	%^a^	*N*	%
At least 1 mosquito net hung	297/664	43.02	422/685	70.1
Children sleeping under mosquito net last night	535/670	80.4	482/684	79.2
Adult sleeping under mosquito net last night	516/665	77.7	469/683	77.8
Carers want to spray the house	642/666	98.0	675/683	98.3
Improved sanitation facilities	460/670	64.8	37/685	3.6
Promptness in seeking care if visiting a CBA	58/90	56.02^**^	6/8	79.9
Promptness in seeking care if visiting CHPS	22/61	36.3	163/228	76.9^**^
Promptness in seeking care if severe malaria signs are identified	55/132	38.2	110/190	64.0
Promptness in seeking care if severe diarrhoea signs are identified	83/179	42.2	212/315	67.4
Promptness in seeking care if severe pneumonia signs are identified	23/52	43.2	116/161	85.4^**^
Adherence to 3 days ACT treatment when visited CBA	16/18	93.2	0/0	0.0
Adherence to CBA ACT prescription	86/90	96.31	8/8	100
CHPS’ staff did not inform about No of days to take ACT	53/61	90.1	201/228	93.5
Adherence to 3 days ACT treatment when visited CHPS	6/6	100	5/7	90.4
Adherence to CHPS ACT prescription	60/61	98.4	220/227	99.28

^a^Weighted estimates

^**^ *p* < 0.05

Predictors of children sleeping under mosquito nets were explored in the univariate and multivariate analysis. Age of child, age of respondent, sex of child, education of respondent, SES, iCCM and CHPS utilization, identifying mosquitos as malaria vectors, and receiving malaria education messages from all different sources were not found to be associated with children under-five sleeping under mosquito nets in the Volta Region. In the Northern Region, the multivariate analysis showed a borderline association between receiving malaria preventive messages from CBAs [adjusted OR = 4.4 (95%CI 0.6, 26), *p* = 0.09] and from friends [adjusted OR = 0.4 (95%CI 0.1, 1.2), p = 0.09] and sleeping under mosquito nets. No other predictors were found (Table 5).

**Table 5:** Predictors of children under- five sleeping under mosquito nets in the Northern Region

Predictors	n/N	%^a^	Adjusted OR (95%CI)	*P*
Receiving malaria messages from CBAs				
No	375/529	79.5	1	0.08
Yes	38/43	93.8	4.3 (0.7, 27.0)	
Receiving malaria messages from friends				
No	342/463	82.8	1	
Yes	71/109	71.6	0.4 (0.1, 1.2)	0.09

^a^Weighted estimates

Only 64.8% and 3.6% of carers in the Volta and the Northern Region reported to have an improved sanitation facility. The association between having an improved sanitation facility and age of respondent, education of respondent, SES, identification of causes of diarrhoea and receiving diarrhoea messages from any source was explored in the Volta Region (there were insufficient numbers to do the analysis on the Northern Region data). Socioeconomic status was found to be associated with having an improved sanitation facility [adjusted OR lower middle SES 5.0 (95%CI 2.0, 12.0), *p* = 0.01; adjusted OR middle SES 3.1 (95%CI 1.0, 9.7), *p* = 0.04; adjusted OR upper middle 7.9 (95%CI 1.8, 34.1), *p* = 0.02; adjusted OR upper 23.8(95%CI 5.0, 112), *p* = 0.007]. Receiving malaria preventive messages from CBAs and from friends each had a borderline association with having an improved sanitation facility [adjusted OR messages from CBAs 1.9 (95%CI 0.8, 4.5), *p* = 0.07, adjusted OR messages from family 0.4 (95%CI 0.2, 1.1), *p* = 0.07]. (Table 6).

**Table 6:** Predictors of having an improved sanitation facility in the Volta Region

Predictors	n/N	%	Adjusted OR (95%CI)	*P*
Socioeconomic status				
Lower	27/133	30.2	5.0 (2.0, 12.0)	0.01
Lower middle	93/132	68.6	3.1 (1.0, 9.7)	0.04
Middle	95/132	64.2	7.9 (1.8, 34.1)	0.02
Upper Middle	114/132	79.0	23.8(5.0, 112)	0.007
Upper	124/132	92.9		
Receiving diarrhoea messages from CBAs			1	0.07
No	304/441	66.0	1.9 (0.8, 4.5)	
Yes	45/55	69.0		
Receiving diarrhoea messages from family			1	0.07
No	322/457	65.3	0.4 (0.2, 1.1)	
Yes	27/39	91.7		

Promptness in seeking care is vital to reduce child mortality and morbidity. iCCM in the Volta Region and CHPS in the Northern Region were associated with prompt seeking care (Table 4). In the Volta Region, 56.0% of carers visiting a CBA did so in the first 24 h of the onset of symptoms while only 36.3% of carers that visited a CHPS did this. When comparing iCCM versus all other appropriate providers (including CHPS, health centres, hospitals, private facilities and licensed chemical sellers), this difference was significant (56.0%, 95% CI 48.7, 63.08 versus 39.4%, 95% CI 29.2, 50.5, *p* = 0.03). The same was not true for CHPS compared to other appropriate providers. In the Northern Region, 79.9% and 76.9% of the carers that visited a CBA and a CHPS did so in the first 24 h of onset of symptoms. In the case of visiting a CHPS versus the other appropriate providers, the differences were significant (77.0%, 95%CI 70.2, 82.7 versus 63.6%, 95%CI 50.2, 75.2, *p* = 0.02). Due to the low number of carers visiting a CBA in the Northern Region, this analysis was not done.

Identifying signs of severe pneumonia was associated with promptness in care seeking in the Northern Region. Eighty five percent of the carers that identified chest in-drawing and noisy breathing as severe signs of pneumonia sought care in the first 24 h of onset of symptoms (*p* = 0.04). No other association was found regarding the identification of severe signs and promptness in seeking care in the Northern Region or in the Volta Region.

Adherence to treatment, following the instructions received from providers, is an important step to obtain optimal treatment outcome. A high proportion of carers reported not being told for how many days they should take ACT treatment. In Volta Region, 45.4% and 90.1% of carers visiting a CBA and a CPHS respectively, and in the Northern Region, 87.1% and 93.5% of carers visiting a CBA and a CHPS reported not being told about the duration of the treatment. A possible reason for this lack of explanation is because a full treatment is packed in one box or envelope. Then, providers often ask users to take the treatment “until it is finished” rather than saying the number of days for which the treatment has to be taken. Of the few carers that were told to take the ACT treatment for 3 days, more than 90% of carers reported having adhered to these 3 days treatment in the Volta and Northern Region when visiting a CBA or a CHPS. Also, when carers were asked if they took the treatment as told, more than 90% of carers in both regions reported to have followed the instructions received in both regions (Table 4).

## Discussion

Three main findings were drawn from this study. Firstly, carers in the Northern Region received more messages than in Volta Region, being malaria messages more common than other messages. These results are similar to the 2008 Ghana Demographic and Health Survey (DHS): 89.8% and 83.3% of carers received malaria messages in the Volta and the Northern Region [19]. Secondly, and in coherence with the information reported on the DHIMS2, more carers in the Volta Region reported to have received malaria, diarrhoea and respiratory illness messages from CBAs and CHPS than the Northern Region [37]. Thirdly, both iCCM and CHPS messages were associated with disease knowledge and behaviour, being health messages from CBAs the predictor associated with a higher number of conditions explored, particularly in the Northern Region. Longer time being exposed to CBA messages (this evaluation came two years and eight years after the implementation in the Volta and Northern Region) could explain these differences between regions. Exposure to health messages from CBAs was found to be associated in the Northern Region with the identification of at least two signs of severe malaria, two practices that can cause diarrhoea, two signs of severe pneumonia, and a borderline association with children sleeping under mosquito nets. Although the association between iCCM utilization and prompt care seeking behaviour could not be explored due to low numbers [16], identifying two signs of severe pneumonia was found to be associated with prompt care seeking behaviour in the Northern Region. In the Volta Region, receiving diarrhoea messages from CBAs had a borderline association with having an improved sanitation facility. In addition, iCCM utilization was associated with prompt care seeking behaviour. Comparing our results with other studies conducted in Ghana, we observed that the 2011MICS, 2008DHS and the 2013LQAS surveys also reported on the carers’ knowledge of the cause of malaria, signs of severe pneumonia, the use of mosquito nets and the prevalence of improved sanitation facilities in Ghana [25, 38, 39]. However, the association between those outcomes and receiving health messages or treatment from CBA or CHPS was not assessed. Studies on the iCCM efficacy or effectiveness did not report on the preventive component- they focused on the therapeutic component of the iCCM strategy. The only study found in Ghana that reported on IEC activity of the iCCM strategy, did not assess the association between the knowledge and behaviour and the source of messages received [38].

Other iCCM studies that reported on carers’ disease knowledge in Mali [40], Uganda [41], Ghana [23], Burkina Faso [23], Ethiopia [23, 42] and Malawi [23] also found similar results. Carers’ in Mali in the iCCM intervention arm were significantly more likely to recognise fever lasting more than one day after treatment and convulsions as danger signs than carers in the control group. In Uganda, more carers in the intervention arm significantly recognised convulsions as a sign to seek care immediately (45%) compared to the non-intervention districts (22%). Carers in Kumasi (Ghana), Lilongwe (Malawi) and Ougadougou (Burkina Faso) improved their knowledge on signs of severe malaria but not those in Bolgatanga (Ghana) and Jimma (Ethiopia) [23]. A longer study (9 years) conducted in Ethiopia showed an increase in the recognition of fast and difficulty in breathing as signs of suspected pneumonia and other childhood danger signs [42]. The contribution of iCCM to prompt treatment seeking behaviour was also found in other studies in Ghana and in Uganda [19, 43, 44].

Health education messages from CHPS’ nurses were associated with the identification of at least two signs of severe diarrhoea in the Volta Region and with promptness in seeking care in the Northern Region. The Multi-Country Evaluation of the IMCI conducted in Tanzania and Bangladesh reported on knowledge of severity of signs and care seeking behaviour [6, 8]. These two studies (a non-randomised controlled trial in Tanzania and a cluster randomised controlled trial in Bangladesh) showed that, when compared with areas without IMCI, carers in the IMCI areas that perceived severity of signs (fast or difficulty in breathing, convulsions, extreme sleepiness, excessive vomiting or inability to drink/breastfeed) were more likely to seek care for their children.

Finally, the similarity of the promotion and prevention messages of iCCM and IMCI, and the association between CBAs’ messages and family and community practices found, suggest that coordination and complementarity between both strategies in Ghana is the logical approach to follow. And therefore, it should be reflected in policy documents and in the organogram of the Ghana Health Service.

### Limitations of the study

This study assessed the effectiveness of the preventive component of the iCCM and CHPS strategy based on the responses of carers of children who were sick in the two weeks preceding the survey. Therefore, the results are not representative of the entire population. In the case of the Volta Region, these results represent the population that had sick children in the previous two weeks in the areas where iCCM and CHPS were implemented (which are rural communities with less access to health facilities).

Families of healthy looking children without fever, diarrhoea or cough could have been included in the study to make appropriate comparison between healthy or sick children. However, this study reports on one component of a broader study to assess the implementation of iCCM and CHPS. The utilisation of both strategies in case of fever, diarrhoea or cough, the effectiveness and cost-effectiveness of the management and the client satisfaction were also addressed. In order to have enough data regarding the management of cases, while having a feasible sample size, we considered that only families of sick children as the study population was the best approach.

Effectiveness was measured based on the association found between the source of message and the desired outcome. We can only conclude that iCCM was a predictor or was associated with better disease knowledge and healthy behaviours. Some potential unidentified confounders could have influenced results.

Carers were asked about health education messages received during the last year on malaria, diarrhoea and respiratory infections. One year was considered good timing to allow a communication strategy to be implemented and to avoid problems in recall. However, recall bias should still be considered, as carers can forget some sources, or remember others that were received more than one year ago. It should be also noted that it is likely that the carers remember the messages that had a higher impact on them. The MICS limit the period of receiving health education to the previous six months before the survey [25]. The DHS did not mention the timing where carers needed to recall health education messages [39]. The other studies reporting on iCCM and CHPS preventive component did not report on sources of health education messages or on the timing for receiving the messages to avoid recall bias: they only report on the improvement of disease knowledge or practice [5, 6, 8–11, 23, 40, 41].

Some of the results had large confidence intervals. A bigger sample size would have been desirable, particularly considering a design effect of 2 instead of 1.5.

## Conclusions

iCCM and CHPS were associated to disease knowledge and health behaviour. Health messages delivered by CBAs were not the most common source of health information, but they were associated with disease knowledge and appropriate health behaviour outcomes, particularly in the Northern Region. This association was more important (in terms of number of outcomes found to be associated with the health message) than that found with CHPS or other health information sources explored. Coordination between iCCM and IMCI could boost the effectiveness of both strategies, both in terms of performance and more efficient use of available resources.

## Additional file

Additional file 1:
**Table S1.** Definitions specific to disease knowledge and health behaviour under HBC and CHPS strategies. **Table S2** Potential predictors explored by outcome. **Table S3** Social and demographic characteristics of respondents. Household survey questionnaire (ODT 108 kb)

## Abbreviations

CBAs: Community-based agents
CHPS: Community-based Health Planning Services
CHW: Community health workers
CI: Confidence interval
DHIMS2: District Health Information System 2
DHS: Demographic health survey
GHS: Ghana health service
HBC: Home-based care
iCCM: Integrated community case management
IEC: Information, education and communication
IMCI: Integrated management of childhood illness
IPTp: Intermittent preventive treatment in pregnant women
IRS: Indoor residual spraying
ITN: Insecticide treated nets
LLIN: Long lasting insecticidal net
LQAS: Lot quality assurance sampling
LSHTM: London School of Hygiene and Tropical
MICS: Multiple indicator cluster survey
NHIS: National health insurance scheme
OR: Odds Ratio
SES: Socio economic status
UNICEF: United Nations Children’s Fund
