# Supplementary material for: Effectiveness of two community-based strategies on disease knowledge and health behaviour regarding malaria, diarrhoea and pneumonia in Ghana
Source: BMC Public Health. 2017 Dec 12;17:948. doi: 10.1186/s12889-017-4964-6 (PMC5727982; doi:10.1186/s12889-017-4964-6)
Supplement: Supplementary file 1 [file 12889_2017_4964_MOESM1_ESM.odt]

| Supplementary Table 1. Definitions specific to disease knowledge and health behaviour under HBC and CHPS strategies |
| --- |
| Definitions | HBC and CHPS strategies |
| Utilization of health services | Realized access or the actual use of the services for seeking health care |
| Knowledge of the cause of malaria | Identification of mosquitoes as vectors of malaria transmission. |
| Knowledge of the causal behaviours/practices of diarrhoea | Identification of at least 2 practices that cause diarrhoea among (i) not washing hands before eating, (ii) not washing hands after defecation, (iii) not drinking clean/boiled water and (iv) having flies on food. No other responses were considered as correct knowledge. |
| Knowledge of the cause of pneumonia | Identification of microorganisms as responsible for the disease. |
| knowledge of causal behaviours/practices of pneumonia | Identification of children not having been vaccinated. No other responses were considered as correct knowledge. |
| Knowledge of signs of severe malaria | Identification of at least 2 signs among (i) unconsciousness, (ii) convulsions, (iii) very pale, (iv) yellow coloured, (v) not breastfeeding/feeding and (vi) vomiting everything. |
| Knowledge of signs of severe diarrhoea | Identification of at least 2 signs among (i) more than 7 days duration, (ii) blood in faeces, (iii) dehydrated and (iv) not breastfeeding/eating. |
| Knowledge of signs of severe pneumonia | Identification of both: (i) chest in drawing and (ii) noisy breathing. |
| Having a mosquito net hanging | At least 1 net over any bed / mat |
|  |  |
| Improved sanitation facility | Flush toilet, pit latrine or ventilated improved latrine |
|  |  |
| Prompt treatment | Care seeking in the first 24 hours of onset of symptoms |
|  |  |
| Adherence to treatment | Following the instructions received from providers on how to take the medicine prescribed. |

| Supplementary Table 2. Potential predictors explored by outcome |
| --- |
| Potential predictors explored |
| Potential predictors of disease knowledge: (i) Age of carers, (ii) education of carers, (iii) socioeconomic status (SES), (iv) receiving information and education messages (on malaria if questions were related malaria knowledge; on diarrhoea if questions related to diarrhoea disease knowledge; on respiratory infections if questions related to respiratory infection knowledge), and (v) receiving malaria education messages from different sources: CBAs, CHPS, health centre, hospitals, friends, family members, neighbours, radio, TV, posters, leaflets and newspapers. Potential predictors of children sleeping under mosquito nets: |
| (i) Age of child, (ii) age of carers, (iii) sex of child, (iv) education of carers, (v) SES, (vi) HBC and CHPS utilization, (vii) identifying mosquitos as malaria vectors, and (viii) receiving malaria education messages from different sources: CBAs, CHPS, health centre, hospitals, friends, family members, neighbours, radio, TV, posters, leaflets and newspapers Potential predictors of improved sanitation facility: |
| (i) Age of carers, (ii) education of carers, (iii) SES, (iv) identification of causes of diarrhoea and (v) receiving malaria education messages from different sources: CBAs, CHPS, health centre, hospitals, friends, family members, neighbours, radio, TV, posters, leaflets and newspapers |

| Supplementary Table 3. Social and demographic characteristics of respondents |
| --- |
|  | Volta Region | Northern Region |
| Indicator | N | % * | N | % * |
| Age |  |  |  |  |
| <20 years | 33 | 3.4 | 25 | 3.9 |
| 20-29 years | 293 | 46.2 | 284 | 44 |
| 930-39 | 226 | 32.1 | 249 | 31.3 |
| >=40 years | 115 | 18.2 | 125 | 19.9 |
| Sex |  |  |  |  |
| Male | 49 | 6.1 | 59 | 9.6 |
| Female | 622 | 93.9 | 626 | 90.3 |
| Marital status |  |  |  |  |
| Single | 44 | 4.1 | 9 | 1.9 |
| Married/co-habited | 574 | 86.2 | 654 | 94.9 |
| Separated/divorced | 37 | 6.9 | 4 | 0.8 |
| Widowed | 16 | 2.7 | 17 | 1.8 |
| Religion |  |  |  |  |
| Christian | 557 | 82.6 | 248 | 52.9 |
| Traditional | 69 | 9.2 | 35 | 6.0 |
| Islam | 28 | 5.4 | 396 | 40.7 |
| Athiest | 11 | 2.5 | 4 | 0.1 |
| Other | 5 | 0.1 | 1 | 0.04 |
| Cannot read in any language | 388 | 65.7 | 579 | 85.3 |
| Education |  |  |  |  |
| None | 258 | 46.6 | 564 | 82.3 |
| Primary | 149 | 21.5 | 59 | 8.4 |
| Middle/secondary | 255 | 30.3 | 53 | 7.8 |
| Technical/Tertiary | 9 | 1.5 | 8 | 1.4 |
| Occupation |  |  |  |  |
| Farming | 358 | 57.4 | 384 | 59.8 |
| Artisan | 60 | 8.7 | 39 | 5.7 |
| Trading | 144 | 21.9 | 109 | 11.1 |
| Government | 12 | 1.9 | 6 | 1.1 |
| Apprentice | 13 | 0.7 | 4 | 0.2 |
| Housewife | 4 | 0.8 | 81 | 13.4 |
| Unemployed | 68 | 7.0 | 41 | 5.7 |
| Other | 11 | 1.4 | 13 | 2.9 |
| Total participants | 671 | 100 | 685 | 100 |
| *Weighted estimates |

Household survey questionnaire

“ASSESSMENT OF THE MALARIA, DIARRHOEA AND PNEUMONIA HOME BASED CARE STRATEGY IN THE VOLTA AND NORTHERN REGIONS OF GHANA”

Household Survey

Questionnaire for caregivers of children under five

2014

The objective of this questionnaire is to collect information on the knowledge of caretakers about the existence of the HMMAD programme at the communities, its utilization and their satisfaction when using the programme. 

Household ID:	

INFORMED CONSENT

Introduction and interview goal: 

Good morning. My name is _________________________________.

I work with the Ghana Health Service. We are here today to discuss your possible participation in a study that will help us improve the health of people in Ghana.

Malaria, diarrhoea and pneumonia are important causes of disease and deaths in your district. The Ghana Health Service started a programme called HOME BASED CARE to help you treat your child with fever and/or diarrhoea at the community, so you do not need to travel long distances and you can get good and quick treatment for your child. We would like to know how this HOME-BASED CARE programme is working at your community and understand how we can improve it so your kids are happy and healthy. For that, we are carrying out interviews with people that have a young child who was sick in the previous 2 weeks.

Procedures: Your participation in this study is entirely voluntary and no one will be penalized if you decide not to participate. If you are willing to participate, we will ask you a few questions on what you do to treat malaria, pneumonia and or diarrhoea. You are free to ask questions and to end this interview at any time. The interview will last approximately 15 to 20 minutes.  

 Risks: This study bears no risk for you or your family members. You may feel uneasy while explaining how you treat malaria, pneumonia and or diarrhoea. Don’t worry, honest answers are important for us and will permit us to know how best the programme can help you to stop malaria, pneumonia and or diarrhoea affecting your child. Please remember that you do not have to answer any questions that make you feel uneasy.

Benefits: If you participate in this study, you will help us find ways to improve access and use of malaria, pneumonia and or diarrhoea treatment services and these interventions can ultimately be used to improve the health of the whole community.    

Confidentiality: Any information discussed will remain strictly confidential. Your name and the name of your family will not appear anywhere. To keep your anonymity, we will use a code rather than your name. We will keep the records in closed files and only the survey personnel will have the permission to access them. Your name and other identifying information will not appear in this study’s report. The results of this interview will be combined with those of at least 580 other respondents.

Right to refuse or to withdraw from the study: As previously stated, you are free to participate in the study or not. If you do participate, you are free to change your mind and decide not to participate further at any time. Your choice to participate in this interview or your refusal to answer some questions will not be told to others. 

Contact Persons: At any moment, if you have questions concerning this study or if you think that this study has caused damage to you, you may contact Mrs. Hannah Frimpong (Tel: 0243235225) or Mrs. Abena Kwaa Addai-Donkoh, (Tel: 0244712919) from the Research and Development Division of the Ghana Health Service. Here is the telephone number to dial if you have any questions regarding your rights related to your participation in this study. (Hand the interviewee the study manager’s and ERC administrator names and telephone numbers).

  

Do you want to participate in this study? [IF YES]: I’m going to read a declaration to you, and if you agree, I will ask you to sign this sheet to confirm your agreement.

  DECLARATION OF CONSENT:  Please sign below once you have read this sheet (or you receive explanations) and:

- You know the reasons and subject of this study

- You know the risks and benefits related to participation in this study 

- AND you have chosen to participate in this study on your own

|  |  |  |  |  |
| --- | --- | --- | --- | --- |
| Name of the interviewee (or legal representative) |  | Signature of the interviewer  (or legal representative) or thumb printing |  | Date |
| Name of witness |  | Signature or thumb printing |  | Date |

I have explained the aim of the project to the interviewee. To the best of my knowledge, this person understands the goal, procedures, risks and benefits of this project.

|  |  |  |  |  |
| --- | --- | --- | --- | --- |
| Name of the interviewer |  | Signature of the interviewer |  | Date |
| Name of witness |  | Signature or thumb printing |  | Date |

NOTE: This consent form, along with the original signature, must be saved in the files by the senior researcher.

FORM ID

Description: !cid_001601c2c2d8$38b13fe0$0100007f@SORYHome Based Care assessment study

Dodowa Health Research Centre

LSHTM

                                                                                                                       

                                                                                          

Unique ID: 

(region code, Interviewer code and serial number): 

| Section 1. IDENTIFICATION (CIRCLE CORRECT ANSWER(S) WHERE CHOICES ARE PROVIDED) |
| --- |
| Interviewer’s Name _______________________________ | Interviewer Code | Date of interview |
| Supervisor’s   Code | Field Editor‘s Code | Data Entry Clerk Code |
| Date | Date | Date |
| District_____________________________________ | District code |  |
| Town/Village___________________________________________ | Town/Village Code |  |
|  | EA code: Cluster number: |  |

| Section 2. CARE GIVER INFORMATION |
| --- |
|  | Is the respondent: | Male Female | 1 2 |  |
|  | How many children under five living in this household had fever OR diarrhoea OR cough/fast-difficult breathing in the past 2 weeks? | Number of children |  |  |
|  | IF MORE THAN 2 SICK CHILDREN UNDER-5 IN HOUSEHOLD, BY LOTERY (Pick 1 from a number of pieces of paper with a number on it linked to a specific child) | Name of the Child |  |
|  | Relation to child NAME | Mother Grand Mother Father Other relative (Specify:______________________________ | 1 2 3 4 |  |
|  | Age of respondent | Age in years |  |  |
|  | Marital status | Single Married Separated Widowed Divorced Co-habited Other Specify….......................................…………… | 1 2 3 4 5 6 7 |  |
|  | What is your highest education level? | Primary JHS/Mid SHS/Sec Techical/Commercial/ Vocacional Tertiary Non Formal education None Other Specify…..................................………………… | 1 2 3 4 5 6 7 8 |  |
|  | Can you read in (MULTIPLE QUESTIONS) | English Ewe Twi Ga Dagbani Fanti Other Specify……………………………………………. No, I cannot read in any language | 1 2 3 4 5 6 7 8 |  |
|  | What is currently your main occupation? | Farming Artisan Trading Government worker Apprenticeship Housewife Unemployed Other Specify…........................…………..…………… | 1 2 3 4 5 6 7 8 |  |
|  | What is the main occupation of the head of household? | Farming Artisan Trading Government worker Apprenticeship Housewife Unemployed Other Specify …....................................……………. Do not know | 1 2 3 4 5 6 7          8           98 |  |
|  | What is your religion? | Christian Traditional African Religion Islam Athiest Other Specify…… …....... …...............……………… | 1 2 3 4 5 |  |

| Section 3: SEEKING CARE FOR FEVER, COUGH/DIFFICULT IN BREATHING OR DIARRHOEA |
| --- |
| I WILL NOW ASK YOU SOME QUESTIONS ABOUT YOUR CHILD (NAME) WHEN HE/SHE WAS SICK |
|  | How old is the child (NAME)? (please, verify with any documentation) | Years Months                           DD        MM        YY Date of birth |  |
|  | Is (NAME) male or female? | Male Female | 1 2 |  |
|  | What signs and symptoms did (NAME) have? (Ask for each of the symptoms) | Fever Chills Sweating Fast Breathing Head ache Body ache Not eating 3 or more stools per day Blood in the stools Cough Noisy breathing Difficulty in breathing Breathing fast Vomiting Very pale skin Unconscious Convulsions Other (specify): ____________________ Not sure | Y 1 1 1 1 1 1 1 1 1 1 1 1 1 1 1 1 1 9 98 | N 2 2 2 2 2 2 2 2 2 2 2 2 2 2 2 2 2 |  |  |
|  | Did you seek care for (NAME)? | Yes 1 No 2 Not sure 98 | IF YES, SKIP TO Q17 |
|  | Why didn't you care for (NAME)? (Multiple responses) | Waiting for fever to lessen Waiting for diarrhoea to lessen Didn't seem ill enough No money for treatment No money for transport No time  Already had medicines in the household Previous bad experience Didn’t know where to go Other Specify: ________________________ Don't know | 1 2 3 4 5 6 7 8 9 10 98 | SKIP TO Q36 |
|  | Where did you first seek care for (NAME)? (1st facility/provider) | CHPS compound Health Centre Hospital Traditional healer Community Based Agent Licensed Chemical Seller Drug peddler Private Health facility Other Specify: ___________________ Don't know | 1 2 3 4 5 6 7 8 9 98 |  |
|  | How long after the fever/diarrhoea/cough or difficulty breathing started did you first seek care from this provider for (NAME)? | Same day Day after Two days after 3 to 7 days More than 7 days Don't know | 1 2 3 4 5 98 |  |
|  | How did you get to this first provider/ facility | Walking Public transport Community assisted transport Ambulance Other. Specify.................................................................. Don’t know | 1 2 3 4 5 98 |
|  | How much time did you spend in travelling to seek care (going and coming back home) at this first facility/provider you visited | Less than 15 min Between 15 min- 30 Between 30 min-1 hour Between 1 and 2 hours Between 2 and 3 hours More than 3 hours Other Specify.......................................................... Don’t know | 1 2 3 4 5 6 7 98 |
|  | For how long did you stay at this first facility/provider you visited (waiting and attending your child) | Less than 15 min Between 15 min- 30 Between 30 min-1 hour Between 1 and 2 hours Between 2 and 3 hours More than 3 hours Other Specify....................................................... Don’t know | 1 2 3 4 5 6 7 98 |
|  | How much money did you spend on travelling to this first provider (going and coming back home) | Cost GHcedis: ____________ Not sure:……………………98 |  |
|  | How much money did you spend buying food while in the facility/provider? | Cost GHcedis: ____________ Not sure:…………………98 |  |
|  | Were there any other costs involved at the facility/ provider (excluding buying drugs)? | Yes 1 No 2 Not sure……………………....….98 | IF NO OR NOT SURE, SKIP TO Q26 |
|  | What costs? | Paying for attending my child………1 Paying for the lab…………………….2 Paying for the NHIS………………….3 Other…………………………………..4 Specify_________________ Not sure…………………………..….98 |  |
|  | Did you seek care for (NAME)’s fever/diarrhoea/cough or difficulty breathing anywhere else? | Yes 1 No 2 Not sure 98 | IF NO OR NOT SURE, SKIP TO Q36 |
|  | Where was the second place that you sought care for (NAME)? (2nd facility/provider) | CHPS compound 1 Health Centre……………..….…...2 Hospital………………………..…..3 Traditional healer…...…………….4 Community Based Agent 5 Licensed Chemical Seller 6 Drug peddler 7 Private Health facility 8 Other 9 Specify: ___________________ Don't know 98 |  |
|  | Why did you seek care elsewhere for (NAME)? | Not getting better 1 Symptoms getting worse 2 To buy/get medication 3 Other 4 Specify: ___________________ |  |
|  | How did you get to this second provider/ facility | Walking Public transport Community assisted transport Ambulance Other. Specify.................................................................. Don’t know | 1 2 3 4 5 98 |
|  | How much time did you spend in travelling to seek care (going and coming back home) at this second facility/provider you visited | Less than 15 min Between 15 min- 30 Between 30 min-1 hour Between 1 and 2 hours Between 2 and 3 hours More than 3 hours Other. Specify................................................................... Don’t know | 1 2 3 4 5 6 7 98 |
|  | For how long did you stay at this second facility/provider you visited (waiting and attending your child) | Less than 15 min Between 15 min- 30 Between 30 min-1 hour Between 1 and 2 hours Between 2 and 3 hours More than 3 hours Other Specify.................................................................... Don’t know | 1 2 3 4 5 6 7 98 |
|  | How much money did you spend on travelling to this second provider (going and coming back home)? | Cost GHcedis: ____________ Not sure:……………………98 |  |
|  | How much money did you spend buying food while in the facility/provider? | Cost GHcedis: ____________ Not sure:……………………98 |  |
|  | Were there any other costs involved at the facility/ provider (excluding buying drugs)? | Yes 1 No 2 Not sure…………………..….98 | IF NO OR NOT SURE, SKIP TO Q36 |
|  | What costs? | Paying for attending my child……..1 Paying for the lab…………………..2 Paying for the NHIS………………..3 Other………………………………....4 Specify_________________ Not sure…………………………..….98 |  |
|  | Did (NAME) take drugs for the fever//diarrhoea/cough or difficulty breathing? | Yes 1 No 2 Herbal medication……………..…3 Not sure 98 | IF NO OR NOT SURE, or herbal medication, SKIP TO Q65 |
|  | Who suggested to take these drugs? | I asked for drugs…………………………….…...1 They were offered by the provider………….….2 Other. Specify………………………………….…3 Not sure……………………………………….…98 |  |
|  | How long after the fever/cough or difficulty in breathing/diarrhoea started did (NAME) take these drugs? | Same day 1 Day after 2 Two days after 3 Three or more days 4 More than 7 days…………...…….5 Don't know 98 |  |
|  | What drugs did (NAME) take for the fever/cough or difficulty in breathing/diarrhoea and from where? (Include any drugs that were from self-medication and/or from a health care provider) Ask if they keep the packages of drugs taken. Show examples of drugs RECORD THE DRUG CODE AND NAME AND FROM WHERE THESE DRUGS WERE TAKEN. Please, see list below: | Drug code Name Facility code* Drug 1 Drug 2 Drug 3 Drug 4 Drug 5 Drug 6 Don’t know 98 |  |
|  | *CODES FOR THE FACILITIES/PROVIDERS CHPS compound ………………………………………………………….1 Health Centre……………………………………………………………...2 Hospital……………………………………………………………………..3 Traditional healer…...……………………………………………………..4 Community Based Agent………………………………………………….5 Licensed Chemical Seller …………………………………………………6 Drug peddler ………………………………………………………………..7 Health facility (private)…………………………………………………….8 Left over drug………………………………………………………….……9 Other ……………………………………………………………………….10 Specify: ________________________________________________ Don't know ………………………………………………………………..98 |  |
| GO THROUGH THE LIST IN Q37 ABOVE AND ANSWER THE NEXT QUESTIONS |
|  | What was the first drug that (NAME) took? (CROSS CHECK WITH Q37) | Drug code: Not sure 98 |  |
|  | How long after the fever/diarrhoea/difficulty breathing began did (NAME) start taking this first drug? | Same day 1 Day after 2 Two days after 3 Three or more days 4 Don't know 98 |  |
|  | How many times a day did (NAME) take this first drug? | Times per day: Not sure 98 |  |
|  | How many tablets or spoons or injections did (NAME) take each time? CIRCLE THE TYPE OF DRUG (1 TEA SPOON=5ML) | Dose each time: Not sure 98 Type: Tablet………………………….1 Tea Spoon……………………2 Injection…………………….…3 Rectal…………………………4 |  |
|  | How many days were you asked (NAME) to take this first drug? | Number of days: Not sure 98 |  |
|  | How many days did you take this first drug? | Number of days: Still taken the drug………………9 Not sure 98 |  |
|  | Did (NAME) take all the prescribed/purchased tablets or syrup of this drug? | Yes 1 No 2 Still taking the drug 3 Not sure 98 |  |
|  | Did you buy the drug? | Yes 1 No 2 Not sure 98 |  |
|  | How much did you pay for this drug? | Cost Ghana Cedis   ________ Not sure 98 |  |
|  | What was the second drug that (NAME) took? (CROSS CHECK WITH Q37) | Drug code: Not sure 98 No other drug……………….1 | IF NO OTHER DRUG, SKIP TO Q65 |
|  | How long after their fever/diarrhoea/difficulty breathing began did (NAME) start taking this second drug? | Same day 1 Day after 2 Two days after 3 Three or more days 4 Don't know 98 |  |
|  | How many days were you asked (NAME) to take this second drug? | Number of days: Not sure 98 |  |
|  | How many days did you take this second drug? | Number of days: Still taken the drug………………9 Not sure 98 |  |
|  | How many tablets or spoons or injections did (NAME) take each time? CIRCLE THE TYPE OF DRUG | Dose each time: Not sure 98 Type: Tablet………………………….1 Spoon…………………………2 Injection…………………….…3 Rectal………………………….4 |  |
|  | Did (NAME) take all the prescribed/purchased tablets or syrup of this drug? | Yes 1 No 2 Still taking the drug 3 Not sure 98 |  |
|  | Did you buy the drug? | Yes 1 No 2 Not sure 98 |  |
|  | How much did you pay for this drug? | Cost Ghana Cedis   ________ Not sure 98 |  |
|  | What was the third drug that (NAME) took? (CROSS CHECK WITH Q37) | Drug code: Not sure 98 No other drug…………….………1 | IF NO OTHER DRUG, SKIP TO Q65 |
|  | How long after their symptoms began did (NAME) start taking this third drug? | Same day 1 Day after 2 Two days after 3 Three or more days 4 Don't know 98 |  |
|  | How many days were you asked (NAME) to take this third drug? | Number of days: Not sure 98 |  |
|  | How many days did you take this third drug? | Number of days: Still taken the drug………………9 Not sure 98 |  |
|  | How many tablets or spoons or injections did (NAME) take each time? CIRCLE THE TYPE OF DRUG | Dose each time: Not sure 98 Type: Tablet………………………….1 Spoon…………………………2 Injection…………………….…3 Rectal………………………….4 |  |
|  | Did (NAME) take all the prescribed/purchased tablets or syrup of this drug? | Yes 1 No 2 Still taking the drug 3 Not sure 98 |  |
|  | Did you buy the drug? | Yes 1 No 2 Not sure 98 |  |
|  | How much did you pay for this drug? | Cost Ghana Cedis   ________ Not sure 98 |  |
|  | Did (NAME) have a blood test for malaria? | Yes 1 No 2 Not sure 98 | IF NO, SKIP TO Q71 |
|  | How was the blood taken? | From the finger/heel…………1 Using a syringe…….….……..2 Not sure……………………...98 |  |
|  | Did (NAME) have the malaria test before taking drugs? | Yes 1 No 2 Not sure 98 |  |
|  | Where did (NAME) get the malaria test done? | CHPS compound 1 Health Centre……...……………...2 Hospital……………………..……..3 Traditional healer…...………..…..4 Community Based Agent 5 Licensed Chemical Seller 6 Drug peddler 7 Private Health facility 8 Other 9 Specify: ___________________ Don't know 98 |  |
|  | What was the result of the malaria test? | Positive 1 Negative 2 Don't know 98 | SKIP TO Q71 |
|  | Why didn’t (NAME) have a blood test for malaria? | Didn’t think fever was malaria ….1 Confident it was malaria ………...2 Don’t have money ……………….3 Don’t know where to get test….. 4 Wasn’t offered one………..……...5 Did not seek care………….……..6 Other ……………………………...7 Specify: ___________________ Don’t know………………………..98 |  |
|  | How is (NAME) now? | Fine, he/she recovered 1 Still sick 2 Ill again/new illness………..……..3 He/she died………………….…...4 Other…………………….………...5 Specify: _______________________ Don't know 98 |  |

Section 4. EXPERIENCE WITH COMMUNITY BASED AGENTS AND OTHER HEALTH PROVIDERS

| THE GHANA HEALTH SERVICE IS TRYIG TO IMPROVE THE TREATMENT GIVEN TO CHILDREN UNDER FIVE. I WILL ASK YOU NOW SOME QUESTIONS ABOUT YOUR EXPERIENCE WHEN CONTACTING COMMUNITY BASED AGENTS AND ANY OTHER HEALTH FACILITY VISITED DURING THIS LAST 2 WEEKS |
| --- |
|  | Have you ever sought care for (NAME) during last 2 weeks? | Yes No Not sure | 1 2 98 | If yes, Skip to Q74 |  |
|  | If no, why you did not go to the CBA this time to seek care for your child’s illness as first option? | I do not know what CBA are There are no CBAs in my community CBA had run out of stock with that drug Cannot afford the CBA drug Does not like the CBA drug Does not like the CBA service The CBA is not competent Prefer to go to the health facility Other Specify.................................................................. Don’t know | 1 2 3 4 5 6 7 8 9 98 | Skip to Q92 |  |
|  | If yes, can I confirm if you went to a Community Based Agent (CBA) to seek care this time for your child’s illness as first option? | Yes No Not sure | 1 2 98 | If no, not sure skip to Q76 |  |
|  | Why did you go to the CBA this time to seek care for your child’s illness as first option? | A relative /friend recommended it Close to me Flexible opening hours That is where I usually go to Competent nurses Competent doctors Better infrastructure Always drugs Other Specify................................................................. Don’t know | 1 2 3 4 5 6 7 8 9 98 |  |  |
|  | Were you satisfied with the service received when you first went to seek care for (Name) illness? | Very satisfied Satisfied Not very satisfied Absolutely not satisfied Not sure | 1 2 3 4 98 |  |  |
|  | If not or absolutely not, why? | Drugs not available Drugs not affordable Drugs not free Have to travel long distances I do not have time to work or to do other things Time to ask for advice and treatment is not flexible Staff not professional Staff is rude Staff do not explain anything  Other Specify………………………………….................... Don’t know | 1 2 3 4 5 6 7 8 9 10 98 |  |  |
|  | If yes or little yes, why? | Drugs readily available Drugs affordable Drugs free Don’t have to travel long distances I have more time to work Flexible time to ask for advice and treatment Professional staff Staff is friendly Staff also explains how to be healthy  Other Specify……………………………........................ Don’t know | 1 2 3 4 5 6 7 8 9 10 98 |  |  |
|  | Do you think the service and treatment received was affordable? | Yes No Not sure | 1 2 98 |  |  |
|  | Did you receive advice on dosage per day? | Yes No Not sure | 1 2 98 |  |  |
|  | Did you receive advice on number of days to take drugs? | Yes No Not sure | 1 2 98 |  |  |
|  | Did you receive advice on side effects (any reaction from the drug)? | Yes No Not sure | 1 2 98 |  |  |
|  | Did you receive advice on what to do if symptoms persist? | Yes No Not sure | 1 2 98 |  |  |
|  | Were you suggested to refer (NAME) to another level of facility? | Yes No Not sure | 1 2 98 | IF NO, SKIP TO Q90 |  |
|  | Why were you suggested to refer (NAME)? | No drugs Child was too sick Child was too many days sick Child was too young to be treated at the community Child was not getting better CBA was not sure about what to do Other Specify…………………………......................... Don’t know | 1 2 3 4 5 6 7 98 |  |  |
|  | Were you given a referral form/card to go to another health facility? | Yes No Don’t know | 1 2 98 |  |  |
|  | Did your child receive an artesunate suppository for the referral (a suppository for malaria)? | Yes No Don’t know | 1 2 98 |  |  |
|  | Did your child receive one dose of amoxicillin for the referral (a medicine for the cough or difficult breathing)? | Yes No Don’t know | 1 2 98 |  |  |
|  | If you were advice to go to another health facility, did you go? | Yes No Don’t know | 1 2 98 |  |  |
|  | Did your child receive a follow up after the first visit? | Yes No Don’t know | 1 2 98 | If no, skip to Q92 |  |
|  | When did your child receive a follow up after first consultancy | The day after Two days after 3 days after Other. Specify………………………………………… Don’t know | 1 2 3 4 98 |  |  |
| I WILL ASK YOU NOW SOME GENERAL QUESTIONS ABOUT CBAs AND HEALTH FACILITIES |
|  | How did you find the time accessibility of the CBAs in your community? | Flexible Not always available Other.Specify........................................................ Not sure | 1 2 3 98 |  |  |
|  | How far is you house from the CBAs | Less than 15 min walking Between 15 min- 30 walking Between 30 min-1 hour walking Between 1 and 2 hours walking Between 2 and 3 hours walking More than 3 hours walking Other Specify................................................................. Don’t know | 1 2 3 4 5 6 7 98 |  |  |
|  | How far is you house from the closest health facility | Less than 15 min walking Between 15 min- 30 walking Between 30 min-1 hour walking Between 1 and 2 hours walking Between 2 and 3 hours walking More than 3 hours walking Other Specify................................................................. Don’t know | 1 2 3 4 5 6 7 98 |  |  |
|  | Which type of facility is the closest to your house | CHPS compound Health Centre District Hospital Regional hospital Private clinic Other: Specify________________________________ Not sure | 1 2 3 4 5 6 98 |  |  |
|  | How did you find the time accessibility at the closest facility? | Flexible Not always available Other Specify…………………............................... Not sure | 1 2 3 98 |  |  |
|  | Where will you go next time if your child is sick again? | CHPS compound Health Centre Hospital Traditional healer Community Based Agent Licensed Chemical Seller Drug peddler Private Health facility Other Specify: _________________________________ Not sure | 1 2 3 4 5 6 7 8 9 98 |  |  |
|  | Do you have an active health insurance | Yes No | 1 2 | If yes, go to Q100 |  |
|  | If no, why? | It is expensive I do not like the service at the health facility I did not know I can be a member I did not know where to go to register I do not think it is necessary The registration is far away from my house It takes too much time to have the NHIS card Other Specify…………………………………………………. Don’t know | 1 2 3 4 5 6 7 8 98 |  |  |

Section 5: KNOWLEDGE OF MALARIA, DIARRHOEA AND PNEUMONIA: SIGNS AND PREVENTION  

|  | What are the signs or symptoms of malaria in a child less than 5 years of age? (Multiple response) | Fever  Chills  Sweating Headache Body ache/Joint pain Fatigue Bitter mouth Loss of appetite Diarrhoea Vomiting Other Specify:_______________ Don’t know 98 | 1 2 3 4 5 6 7 8 9 10 11 98 |  |
| --- | --- | --- | --- | --- |
|  | What signs and symptoms make you decide that malaria is serious in a child less than 5 years of age? (Multiple response) | Unconscious Convulsions Fast breathing Very hot Yellow eye colour Very pale skin Not breastfeeding Not eating Frequent vomiting Diarrhoea Vomiting Other Specify:__________________ Don’t know 98 | 1 2 3 4 5 6 7 8 9 10 11 12 98 |  |
|  | How do people get malaria? (Multiple response) | Mosquito bites Drinking dirty water Not boiling water Bathing in river Bad air Bad talking Spirits Bad food Poor hygiene Other Specify:__________________ Don’t know | 1 2 3 4 5 6 7 8 9 10 98 |  |
|  | In the past year have you seen or heard any messages about malaria (how it is transmitted, how to avoid it, symptoms and what to do when sick? | Yes No Not sure | 1 2 98 | If not  skip to Q105 |
|  | From where did you hear messages about malaria?    (Multiple response) | Neighbour Family member Friend Community Based Agent (CBA) Nurse from CHPS compound Nurse from Health Centre At the hospital Radio TV Posters Leaflets Newspaper Other Specify:____________________ Don’t know 98 | 1 2 3 4 5 6 7 8 9 10 11 12 13 98 |  |
|  | Did [Name of child] sleep under mosquito net last night? | Yes No Not sure | 1 2 98 |  |
|  | There is at least 1 mosquito net hanged up in the house? (VERIFY IF THERE IS AT LEAST 1 MOSQUITO NET HANGED UP) | Yes, I have seen it No, I have not seen it Not allowed to check | 1 2 3 |  |
|  | Did you sleep under a mosquito net last night? | Yes No Not sure | 1 2 98 |  |
|  | Do you know what indoor residual spraying is? | Yes No Not sure | 1 2 98 |  |
|  | Would you be happy to have your house sprayed with insecticide to kill mosquitoes? | Yes No Not sure | 1 2 98 |  |
|  | Has your house ever been sprayed with insecticide to kill mosquitoes? | Yes No Not sure | 1 2 98 |  |
|  | What are the signs or symptoms of severe diarrhoea in children less than 5 years of age? (Multiple response) | Diarrhoea present for more than 7 days Diarrhoea with blood in stools Dehydrated, dry mucosa Headache Fatigue Not breastfeeding, not eating Other Specify:___________________ Don’t know 98 | 1 2 3 4 5 6 7 98 |  |
|  | Which practices in the community can cause diarrhoea in children less than 5 years of age? (Multiple response) | Not cleaning hands before food preparation Not cleaning hands before eating Not cleaning hands after defecation Not boiling water for drinking/cooking Flies on food The sun Bathing in river Bad air Bad talking Spirits Bad food Other Specify____________ Don’t know | 1 2 3 4 5 6 7 8 9 10 11 12 98 |  |
|  | In the past year have you seen or heard any health messages about diarrhoea? | Yes No Not sure | 1 2 98 | If not  skip to Q115 |
|  | From where did you hear messages about diarrhoea?    (Multiple response) | Neighbour Family member Friend Traditional healer Community Based Agent Nurse from CHPS compound Nurse from Health Centre At the hospital Radio TV Posters Leaflets Newspaper Other  Specify:_______________________ Don’t know | 1 2 3 4 5 6 7 8 9 10 11 12 13 14 98 |  |
|  | What are the signs or symptoms of severe acute respiratory infection? (Multiple response) | Duration of symptoms (number of days with symptoms) Breathing quicker than usual Chest in-drawings Noisy breathing Other Specify………………………………………… Don’t know | 1 2 3 4 5 98 |  |
|  | What can cause respiratory infections in children less than 5 years of age? (Multiple response) | Infections (microorganisms) Not cleaning hands Not boiling water Bathing in river Being close to a person that coughs No vaccinating my child Bad talking Allergies Spirits Bad food Poor hygiene Other Specify________________________ Don’t’ know | 1 2 3 4 5 6 7 8 9 10 11 12 98 |  |
|  | In the past year have you seen or heard any messages about respiratory infections? | Yes No Not sure | 1 2 98 | If not  skip to Q119 |
|  | From where did you hear messages about respiratory infections? | Neighbour Family member Friend Traditional healer Community Based Agent Nurse from CHPS compound Nurse from Health Centre At the hospital Radio TV Posters Leaflets Newspaper Other Specify:________________________ Don’t know | 1 2 3 4 5 6 7 8 9 10 11 12 13 14 98 |  |

| Section 6:  HOUSEHOLD CHARACTERISTICS |
| --- |
|  | Do you (or the household head) own or rent your home? | Own Rent Squatters Care takers Rent free Other Specify………………………...................... Not sure | 1 2 3 4 5 6 98 |  |
|  | How many rooms are in your household? | Number of Rooms Don’t know    98 |  |
|  | What is the main material used for flooring in your house? Interviewer’s observation | Earth/Sand/Mud/Dung Wood Planks Palm / Bamboo Parquet or Polished Strips Ceramic Tiles Cement Other Specify…………………..........…………… | 1 2 3 4 5 6 7 |  |
|  | Does your house hold have any of the following assets? Read out the list. (MULTIPLE RESPONSE) | Electricity Radio Television Refrigerator Telephone | Y 1 1 1 1 1 | N 2 2 2 2 2 |
|  | Does any member of your household own? (MULTIPLE RESPONSE) | Bicycles Motorcycles Car Canoe Tractor Other. Specify……………………………..……... | Y 1 1 1 1 1 | N 2 2 2 2 2 |
|  | What is the main source of drinking water for members of your household? | Piped Water Residence Piped Water in Public Taps Household well Public Well Borehole River, canal or Surface water Rainwater Tanker truck Water sachets Other Specify……………….......………………... | 1 2 3 4 5 6 7 8 9 10 |  |
|  | What type of toilets does your household use? | Flush toilet Pit Latrine Ventilated Improved pit latrine Bucket / Pan latrine Bush/ Field as latrine Other Specify……………………...................…… | 1 2 3 4 5 6 |  |
|  | Is your toilet is ‘shared by other households? | Yes No | 1 2 |  |
|  | What do you use as your main source of fuel for cooking in this household? (Multiple answers) | Gas Electricity Charcoal Wood Other Specify……………….......………………… | 1 2 3 4 5 |  |
|  | How many people currently live in your household? Include permanent residents and temporary visitors. | Number of people in household  Not sure     98 |  |  |

Thank you very much for your time
